# Children with lower language skill engage additional brain mechanisms in both hemispheres during sentence comprehension

**DOI:** 10.21203/rs.3.rs-7871439/v1

**Published:** 2025-10-21

**Authors:** Marjolein Mues, Avantika Mathur, James R. Booth

**Affiliations:** Vanderbilt University; Vanderbilt University; Vanderbilt University

## Abstract

Multi-voxel pattern analysis was used to determine whether a support vector machine (SVM) could distinguish between grammatically correct sentences and sentences with a morphological violation related to tense-marking in 7-year-old children (N = 92) undergoing functional magnetic resonance imaging. SVM classifier accuracy was examined in regions of interest (ROIs) related to phonological processing (inferior frontal gyrus, pars opercularis and posterior superior temporal gyrus) and semantic processing (inferior frontal gyrus, pars triangularis and posterior middle temporal gyrus) determined with independent functional localizers using sound and meaning judgments. In these ROIs, the classifier performed around chance indicating that grammatical sentences and sentences with a morphological violation have a similar underlying representation for the whole sample. We also investigated correlations of standardized measures of language skill with classifier accuracy in the ROIs and in the whole brain. Whole brain analyses showed lower skill was associated with higher classification accuracy in bilateral brain areas implicated in semantic processing, in articulation and phonological processing and in verbal working memory. Overall, children with lower language skill seem to use widespread mechanisms in both hemispheres for sentences comprehension, possibly indicating increasing use of rehearsal mechanisms and semantic compensation for detecting morphological errors.

## 1. Introduction

Morphological processing is a key linguistic feature that refers to the processing of the smallest possible linguistic units that carry meaning. These linguistics units, called morphemes, convey grammatical information, such as tense and agreement with the subject. For example, by adding either the morpheme /-ing, /-s or /-ed, we can indicate whether someone is talking now, always talks, or did so yesterday. Morphological processing skills are related to both children’s phonological segmentation abilities e.g., ^1^ and semantic facets of language such as vocabulary development e.g., ^2^. Morphological processing skills facilitate subsequent language and reading abilities ^3,4^. Difficulties in processing morphemes, especially in marking tense, are prevalent in children with language difficulties, including those with developmental language disorder (DLD)^5^.

As morphological operations are by definition performed on single words, previous scientific studies have mainly examined morphological processing at the word level^6–8^. Distributed accounts of morphological processing in words describe morphemes as representing the mapping between form (phonology) and meaning (semantics)^9,10^. Within this framework, the development of morphological processing involves learning the regularities between form and meaning. As the relationships of phonology and meaning to morphology may differ depending on the context, processing morphology in single words may be easier than in sentences^11,12^, so results at the word level may not fully apply to sentences.

Previous studies at the word-level and at the sentence level have investigated whether morphological processing may rely more on phonological or semantic mechanisms. Phonological mechanisms may be primary in their support of morphological processing as theories of sentence processing pose that phonological decoding takes place before meaning can be assigned^13–15^. Phonemic segmentation occurs rapidly, within 100 milliseconds of hearing a speech stream, while semantic encoding appears to happen later, i.e. at about 300–500 milliseconds post stimulus onset see for an overview of event-related potential (ERP) evidence ^16^. Phonological mechanisms thus have a temporal advantage over semantic mechanisms. Our previous work examining neural mechanisms underlying morphological processing of auditory sentences in seven-year-old children shows that areas related to phonology, specifically the Inferior Frontal Gyrus – pars opercularis (IFGop) and posterior Superior Temporal Gyrus (pSTG), were activated during an in-scanner grammaticality judgement task requiring the detection of morphological errors^17^. These regions comprise the dorsal stream for language involved in phonological processing, namely, the pathway involved in sound to articulation mapping^18,19^. In addition to the dorsal stream, the ventral stream, including the Inferior Frontal Gyrus – pars triangularis (IFGtri) and posterior Middle Temporal Gyrus (pMTG) is considered to be a pathway for mapping sound to meaning^18,20^. In the ventral pathway, the same study on seven year olds showed activation within the pMTG during the in-scanner grammaticality judgement task, though the cluster was smaller in size than those in the dorsal areas^17^.These fMRI results showing the relative importance of phonology are supported by Louleli et al.^21,22^ who showed, using magnetoencephalography (MEG) in seven-year-olds, that top-down processes based on long-term phonological representations were especially relevant for processing of morphological features in a sentence context.

The relative reliance on phonological and semantic mechanisms during morphological processing may depend on skill. Adults who have less effective phonological mechanisms rely more on semantic engagement when processing morphologically complex words compared to those with intact phonological skills^7^. Although this overreliance on semantic engagement can result in successful morphological processing, participants exhibit longer response times, indicating that this strategy is less effective than relying more on phonological representations. Similarly, children with low language skill, including those with Developmental Language Disorder (DLD) who experience challenges with phonological processing but show a relative strength in semantic skills, show an overreliance on semantic mechanisms that in turn results in less effective morphological processing^5,23,24^. For example, in children with DLD, auditory processing of morphemes in sentences improves when the articulation rate is slowed^25^, indicating that facilitating phonological processing allows for better morphological processing. A study examining school-aged Chinese speaking children using electroencephalography (EEG) reported that children with DLD show a sub-optimal yet robust over-reliance on semantic mechanisms during processing of morphosyntactic errors in sentences relating to classifier-noun agreement^26^. Specifically, children with DLD showed an enhanced N400 Event-Related Potential (ERP), which is related to semantic processing, whereas typically developing children showed the expected P600, an ERP related to morphosyntactic processing^26^.

The over-reliance on semantic mechanisms for morphological processing in the absence of effective phonological processing in children with low language skill is in line with research in clinical groups, such as in aphasia and dyslexia. One study examining agrammatic aphasic patients using EEG reported that patients listening to sentences with a morphosyntactic violation showed semantic-related ERPs while age-matched healthy controls showed morphosyntax-related ERPs, providing evidence of semantic compensation^27^. In dyslexia, studies show that both children and adults access semantic mechanisms to compensate for decreased phonological abilities during reading^28–30^. Even in non-clinical populations, children and adults that are less skilled readers show weaker phonological processing and a stronger reliance on lexical-semantic pathways during reading, suggesting that semantic knowledge can compensate for phonological weakness^31,32^. It has been suggested that such a semantic compensatory mechanism may not only exist for written language, but also for spoken language given the high rate of comorbidity between dyslexia and DLD^32^. Thus, semantic compensation to process morphosyntactic linguistic features appears to be widespread in those with lower language skill who are less able to rely on phonological mechanisms.

In our previous study, we did not find correlations between language skill and morphological processing in sentences within the dorsal stream implicated in phonological processing or within the ventral stream implicated in semantic processing but whole brain exploratory correlations were revealed within the cerebellum^17^. These correlations showed a negative relationship between activation in the cerebellum during a grammaticality judgement task and general language abilities, indicating that those with lower language skill engaged the cerebellum to a greater degree. Because the area of the cerebellum related to language skill is involved in phonological and phonemic processing, we hypothesized that lower skill language learners may engage this area to aid in phonological processing, enabling them to avoid using semantic strategies for processing morphemes^17^. However, the univariate approach of this study may not have been sensitive enough to detect subtle relationships of language abilities to morphological processing within the dorsal and ventral regions of interest (ROIs). In the current study, we aim to further explore whether language abilities are related to morphological processing using multi-voxel pattern analysis (MVPA) with a searchlight approach. A main benefit of MVPA is increased sensitivity as the analysis examines distributed patterns of activity^33,34^. By looking at the contributions of multiple voxels using pattern-classification techniques, signal-loss issues that arise from spatially averaging across voxels in univariate approaches is avoided. We trained Support Vector Machines (SVM) to learn to distinguish between different experimental conditions on partial data and then tested on new data using the leave-one-trial-out-cross-validation method. SVM was trained and tested on the ability to classify sentences that have a morphological violation compared to grammatically correct sentences. We also determined whether these classification accuracies were related to general language and morphological processing skill.

Because we were interested in the role of phonological and semantic mechanisms in morphological processing, we used independent localizers to identify ROIs in the dorsal and ventral pathways. In addition, we examined whether skill was related to classification accuracy across the whole brain. Based on previous research indicating that phonological strategies are more advantageous for morphological processing^7,17,22^, we hypothesized that children with higher language skill would rely more on phonological mechanisms for morphological processing. This would result in significant positive correlations between language abilities and classifier accuracy in the dorsal pathway (i.e., IFGop and pSTG). We expected that children with lower language skill may rely on semantic mechanisms, resulting in significant negative correlations between language abilities and classifier accuracy in the ventral pathway (i.e., IFGtri and pMTG). The hypotheses and analysis plans of this study were preregistered through the Open Science Framework after verifying participant eligibility and the creation of the functional ROIs, which were used in a previous project^17^, but prior to the MVPA data analysis. The preregistration is available at https://osf.io/6vdaw.

## 2. Results

Standardized scores for the Sentence Repetition and Word Structure tests and accuracy scores for the in-scanner tasks are shown in Table 1.

### 2.1. ROI results

MVPA searchlight analyses revealed at-chance (~ 50%) results within each of the ROIs. None of the classification accuracies significantly distinguished between grammatical sentences and sentences with a finiteness violation. We also did not observe significant correlations between the accuracy of classifiers in any of our functional ROIs and the Sentence Repetition or Word Structure tests.

### 2.2. Whole brain results

The preregistered whole brain partial correlation analyses revealed significant negative correlations between classification accuracies and behavioral scores on both Sentence Repetition and Word Structure. For correlations with Sentence Repetition, examination in bspmview (https://www.bobspunt.com/software/bspmview/) indicated that these results were mostly located within the left hemisphere in the frontal language areas, especially the IFG triangularis. A summary of the locations of significant correlations with Sentence Repetition larger than k = 10 voxels is given in Table 2. The full table can be found in Supplementary Table 1. Correlations with Word Structure were especially present in the right hemisphere, mostly in the frontal lobe but also across other areas. A summary of the locations of these correlations with larger than k = 10 voxels is given in Table 3. The full table can be found in Supplementary Table 2. To simplify interpretation, we chose to only display clusters with more than 10 voxels in Tables 2 and 3, even though with TFCE a single voxel with enough signal intensity can remain significant. If one brain area had multiple single voxels that together made 10 or more, they are displayed under a single anatomical label in Tables 2 and 3. Coordinates are taken from the largest cluster. For example, in Table 2, the left calcarine gyrus is displayed as 10 voxels. As can be seen in Table S1, the results consist of a cluster of 6 voxels, a cluster of 2 voxels and 2 single significant voxels within this area. The z-value is averaged across the voxels ((6*z-value cluster of 6 + 2*z-value cluster of 2 + z-value of single voxel + z-value of single voxel)/10).

**Figure 1:**
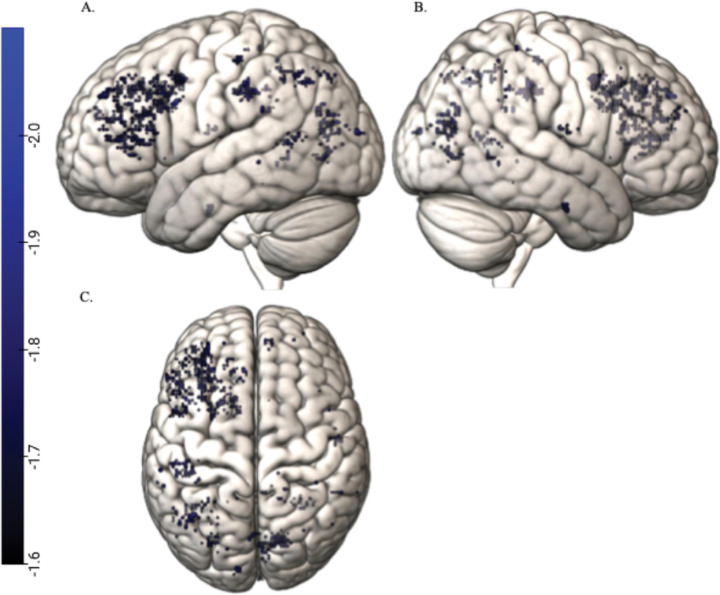
Negative whole brain correlations between Sentence Repetition and search light accuracies surviving TFCE corrections. Note: Figure shows the TFCE corrected negative whole brain correlation between Sentence Repetition and the whole brain search light accuracies. Panel A shows the negative correlations surviving TCFE corrections for multiple comparisons in the left hemisphere, panel B for the right hemisphere and panel

**Figure 2:**
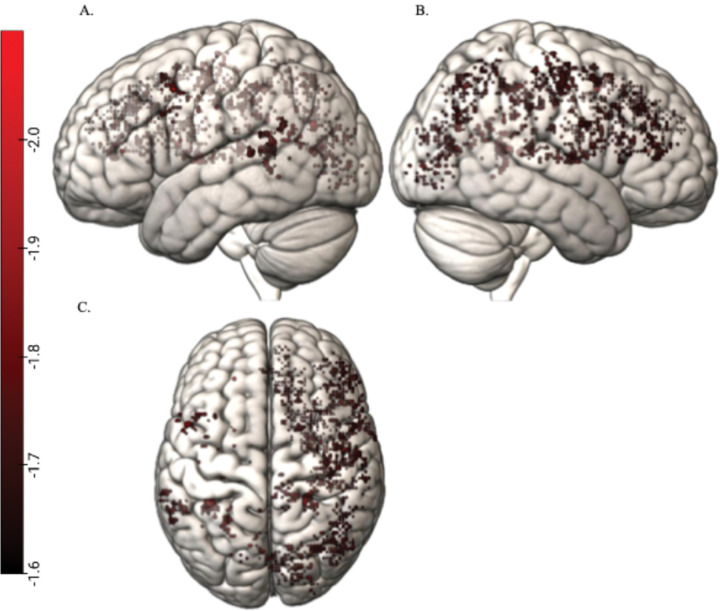
Negative whole brain correlations between Word Structure and search light accuracies surviving TFCE corrections Note: Figure shows the TFCE corrected negative whole brain correlation between Word Structure and the whole brain search light accuracies. Panel A shows the negative correlations surviving TCFE corrections for multiple comparisons in the left hemisphere, panel B for the right hemisphere and panel C shows a superior view (left side representing the left hemisphere). The color bar shows the z-values for the voxels with correlation values surviving TFCE corrections (z ≤ −1.64).

Figure 1 shows the correlations for Sentence Repetition and Figure 2 shows these correlations for Word Structure.

## 3. Discussion

The main question of this study was whether phonological and semantic mechanisms were engaged during the grammaticality judgment of auditorily presented sentences, and whether this engagement was related to language skill. More specifically, we determined whether a support vector machine (SVM) using searchlight multivoxel pattern analysis (MVPA) could distinguish between grammatical sentences and sentences with a morphological violation and whether this classification accuracy was related to language skill. A novel aspect of our work was the use of rhyming and meaning tasks to independently identify these mechanisms in regions of interest (ROIs) in a dorsal pathway implicated in phonology, including the inferior frontal gyrus opercularis (IFGop) and posterior superior temporal gyrus (pSTG), and a ventral pathway implicated in semantics, including the inferior frontal gyrus triangularis (IFGtri) and posterior middle temporal gyrus (pMTG). We also examined brain-behavior correlations with language skill at the whole brain level. Our hypothesis was that lower language skill would be associated with the compensatory use of semantics due to less effective engagement of phonology when processing sentences. Overall, we did not find effects in our ROIs, but the whole brain analyses show that lower skill was associated with widespread engagement of bilateral regions.

Our initial MVPA results showed that the classifier could not accurately distinguish between grammatical sentences and sentences with morphological violations within our four ROIs. This is not in line with a previous univariate examination on the same sample that showed clusters of activation in the IFGop, IFGtri and pSTG for the contrast of sentences with a finiteness violation > grammatical sentences, indicating that activation within these ROIs was greater for sentences with morphological errors^17^. MVPA exploits voxel-level variability within subjects that is not considered in univariate analyses and discards mean activation variability between subjects. It is this between subject-level variability in mean activation that can lead to reduced sensitivity in univariate analyses^35^. Given the higher sensitivity of MVPA over univariate analyses^35^, the absence of effects in the MVPA results in the presence of effects in the univariate analyses is intriguing. While it is not possible to directly compare the previous univariate results to these new MVPA results, the lack of MVPA results suggests that the underlying representations for sentences with and without morphological errors is either shared or similar, resulting in failure to reliably distinguish between conditions in these areas. The greater activation in the IFGop, IFGtri and pSTG during the morphological condition compared to the grammatical condition in the univariate analyses may indicate that although the same representations are activated in the two conditions, they are activated more robustly in sentences with grammatical violations. This could suggest a highly efficient neural process where errors are detected by the modulation of a single underlying representation rather than recruiting an entirely different representation.

We next examined brain-behavior correlations between the classification accuracies and language skill both within our ROIs and in the whole brain. We examined general language skill using the Sentence Repetition test, in which participants simply had to repeat a sentence that they heard, and we examined more specific morphological language skill using the Word Structure test, in which children needed to produce appropriate morphemes. We did not observe brain-behavior correlations surviving TFCE corrections for multiple comparisons within our ROIs but the whole brain analyses revealed negative correlations outside our ROIs in the left hemisphere and in widespread regions in the right hemisphere. Overall, these results indicate that children with lower language skill show more distinct representations for grammatically correct sentences compared to sentences with morphological errors than those with higher language skill. This finding is in line with our previous univariate results that showed engagement of additional brain areas, specifically the cerebellum, for children with lower language skill ^17^.

Correlations with language skill in the left hemisphere were found in the frontal cortex, especially within the left IFGtri, which has been associated with semantic processing^18^. Lower language skill was also associated with higher classification accuracy in the left angular gyrus and in the left middle temporal gyrus (MTG), both of which have been implicated in semantics^36–38^. In the Introduction, we reviewed evidence that those with dyslexia^28–30^ and DLD^26^ may compensate with semantic involvement due to their deficient phonological processing. Our results are consistent with the argument that children with lower skill may additionally rely on semantic mechanisms in the left frontal, parietal and temporal cortex.

Lower language skill was associated with higher classification accuracy in widespread regions of the right hemisphere. Although language is left lateralized in most individuals, the right hemisphere plays an important role^39–41^. Whereas the left hemisphere seems to be more important for local details, the right hemisphere is thought to be especially involved in global processing required by semantics or discourse context^39,42,43^. Studies have shown that global or distant semantic relationships engage the right frontotemporal regions, whereas the left hemisphere appears to preferentially process local or close semantic relationships^44–48^. In our study, children with lower language skill may differentially engage sentences with and without grammatical errors in the right hemisphere because of their increased reliance on distant semantic relations to determine plausibility. We find language skill is related to large numbers of voxels in the IFG triangularis and the angular gyrus in the right hemisphere, both of which are implicated in semantic processing^49^.

Curiously, we also find lower language skill is related to better classification accuracies in bilateral regions in the occipital cortex, especially the Calcarine Gyrus and the Lingual Gyrus. Previous studies show that sentence processing activates the visual cortex ^50^. This may especially be the case for children compared to adults, potentially because children use an imagery strategy rather than a linguistic strategy to understand sentences^51^, though whether this strategy is the reason for the activation is debated^50^. There is emerging evidence that the occipital cortex, especially the lingual gyri, may be part of a semantic association network facilitating language comprehension^52,53^, pointing towards semantic compensation in those with lower skill.

We thought that children with lower language skill may rely less on phonological mechanisms, but we showed lower language was associated with higher classification accuracies in regions implicated speech processing, namely the left Superior Temporal Gyrus (STG)^18,54^. To extend to this, we find significant negative correlations in the bilateral Pre- and Postcentral Gyrus and in the right Rolandic Operculum. The Precentral Gyrus is directly related to speech perception and phonological processing^55,56^ while the Postcentral Gyrus is known for its role in the integration of sensory information^57^ and the Rolandic Operculum is implicated in sensory and motor processing^58^. All of these structures play a role in speech perception, planning and articulation^59–61^. We also found that lower language skill was associated with higher classification accuracies in the insula and basal ganglia. The insula and basal ganglia have been hypothesized to play a role in articulation and phonemic processing, either directly or indirectly through relationships with cortical structures^62–66^. A recent meta-analysis of Ullman et al.^67^ implicates anomalies in the basal ganglia in children with DLD. We hypothesize that lower skill children may rely on these regions for morphological processing by covertly or overtly rehearsing the sentences. This reliance on internal rehearsal is additionally supported by the finding that lower language skill was related to large clusters in bilateral middle frontal gyrus (MFG). The MFG has been implicated in various working memory tasks^68,69^. Increasing the cognitive load of working memory tasks results in greater activation in MFG to cope with the enhanced demand^70–72^. Children with lower language skill may experience an increased cognitive load compared to children with higher language skill when they are making a grammaticality judgement. This is in line with a previous meta-analysis showing that children with DLD experience working memory deficits^73^.

Our findings show that children with lower language skill rely on additional brain mechanisms during sentence processing, indicating potential compensatory mechanisms. However, it is also true that those with higher language skill rely on fewer brain mechanisms, which is consistent with the debated Neural Efficiency Hypothesis. This hypothesis broadly posits that those with higher intelligence show more “efficient” brain functioning, resulting in lower functional activation, depending on task demands^74–76^. As we do not directly examine neural activation, our results cannot directly provide data to support this hypothesis, but our findings indicate that individual differences in skill play a role in neural processing.

In this study, we used MVPA to examine the nature of the representations of grammatical sentences and sentences with a morphological error. Our results for the sample as a whole point towards a shared or similar underlying representation for these two sentence types. However, we show that lower skill children rely on a network extending beyond the typical left-hemispheric frontotemporal language areas in bilateral brain regions. The results are consistent with the use of semantic compensatory mechanisms to process sentences in children with lower language skill, but they also seem to have altered phonological mechanisms.

## 4. Method

### 4.1. Participants

Participants were selected from an open-access dataset on OpenNeuro (https://openneuro.org/datasets/ds003604/versions/1.0.7) titled “A longitudinal neuroimaging dataset on language processing in children ages 5, 7, and 9 years old”^77^. This dataset has longitudinal data at three time points, namely when children were five years old, seven years old and nine years old. The current study utilizes data from the second time point when children were seven years old (*n* = 294). No new data was collected, all analyses performed in this study relied entirely on the publicly available dataset on OpenNeuro. After applying motion and accuracy criteria (see section 2.3) for the tasks of interest, the sample included 105 children. After examining behavioral inclusion criteria, the final analytic sample comprised 92 participants (37 male, 55 female) between 7.0 and 8.3 years old (M = 7.4, SD = .3). Behavioral inclusion criteria were: 1) mainstream English speakers, defined as scoring 9 out of 15 on the Diagnostic Evaluation of Language Variation (DELV) Part 1 Language Variation Status^78^; 2) primarily right-handed defined as performing at least 3 out of 5 tasks out of writing, drawing, picking-up, opening and throwing items using their right hand; 3) obtaining a score of > 70 on the Kaufman Brief Intelligence Test, Second Edition (KBIT-2)^79^, 4) having no clinical diagnosis of neurological, psychiatric, or developmental disorders as reported in a parent questionnaire.

Parents/legal guardian(s) completed informed consent for their children prior to testing. Verbal assent was collected from participants prior to testing. The study procedures were approved by the Institutional Review Board of the University of Texas, Austin (protocol number 2014–07-0018). All methods were carried out in accordance with relevant guidelines and regulations.

### 4.2. Behavioral assessments

#### Language abilities

We included two standardized measures in our brain-behavior analyses to examine if the role of phonological and semantic mechanisms during sentence processing is related to general language ability or morphological language skill. General language ability was characterized by the Sentence Repetition test of the Clinical Evaluation of Language Fundamentals, fifth edition (CELF-5)^80^. We chose Sentence Repetition as a proxy of general language ability as it is perhaps the best reflection of various underlying linguistic processes at different levels including speech perception, lexical knowledge, grammatical encoding^81^ and does not seem to disproportionally rely on either phonological or semantic skills. Morphological skill was measured with the Word Structure test of the CELF-5. This test elicits different morphemes by asking questions such as “here is one girl, here are two …?”. The child is then supposed to answer with the target word and add the correct morpheme, in this case answering: “girls”.

#### Nonverbal cognitive abilities

The Kaufman Brief Intelligence Test, second edition (KBIT-2)^79^ was used to assess nonverbal IQ. All children in the final sample scored greater than or equal to a standard score of 70. Nonverbal cognitive abilities standard scores were included in all brain-behavior analyses as a covariate of no interest.

### 4.3. fMRI tasks

Children were invited for a practice session in a mock scanner to get familiar with the in-scanner tasks and the scanning environment prior to the MRI visit. During their actual MRI visit, T1-weighted and task-based fMRI sequences were collected. Data for four fMRI tasks were collected, three of which were analyzed for this paper: a grammaticality task, a sound task and a meaning task. These tasks have previously been described in papers relating to the same dataset, including Mues, et al. ^17^. For each task, participants completed two runs. Participants typically completed all four tasks (eight runs) across two days, with each session lasting around an hour. Participants responded to stimuli through button boxes and were instructed to respond as quickly and accurately as possible by using their right index finger to respond “yes” and their right middle finger to respond “no” on a button box.

The grammaticality task consisted of an auditory sentence judgement task. Children were asked to indicate if “the way she speaks sounds right”, eliciting a yes or no response. The task had three experimental conditions: a grammatically correct condition, a plurality violation condition and a finiteness violation condition. Children also completed a perceptual control condition for which they were instructed to press the “yes” button when they heard two frequency modulated white noise sounds.

Table 4 shows example sentences of each condition. Each of the two runs contained 40 trials (i.e., 80 trials in total), with ten trials per condition per run. Stimuli duration ranged between 2740 and 4972 ms and was followed by a 1234 to 4611 ms jittered response interval. The length of trials (5174 to 8422 ms) was equated across conditions. In this study, we trained and tested the classifier to distinguish between the finiteness violation condition with the grammatically correct condition to characterize morphological processing.

The sound task consisted of an auditory word-level phonological judgement. Participants heard two sequentially auditory one-syllable words and had to indicate whether the two words “shared any sounds”, eliciting a yes or no response. The task had three experimental conditions: a rhyme condition, an onset condition where only the first sound was shared between the two words, and an unrelated condition, see Table 5 for example stimuli. Note that this Table was derived from previous papers describing the stimuli, including Mues, et al. ^17^. The children also completed the perceptual control condition consisting of frequency modulated noise. The task included 48 trials per run (i.e., 96 runs in total), with each run containing 12 trials per condition and lasting around three minutes. Each word had a duration ranging from 439 to 706 ms, and the second word was presented approximately 1000 ms after the onset of the first word. The total stimuli duration per trial ranged from 1490 to 1865 ms and was followed by a jittered response interval that varied between 1500 and 2736 ms.

The meaning task consisted of an auditory word-level semantic judgement. Just as for the sound task, participants would hear two sequentially presented one-syllable words, but for this task they were asked if the words “go together”. The task had three experimental conditions: a high association condition, a low association condition and an unrelated condition, see Table 5 for examples. Children also completed the perceptual control condition, consisting of frequency modulated noise. The semantic task included 48 trials per run (96 runs in total), with each run containing 12 trials per condition and each run lasting around three minutes. Each word had a duration ranging from 500 to 700 ms, and the second word was presented approximately 1000 ms after the onset of the first word. The total stimuli duration per trial ranged from 1500 to 1865 ms and was followed by a jittered response interval that varied between 1800 and 2701 ms. More specifics of the fMRI tasks can be found in Wang, et al. ^77^. The sound and meaning tasks were used to create functional localizers in which the search light analysis was conducted.

#### Accuracy and motion criteria

Participants with acceptable task accuracies who did not show a response bias were included in the analyses to ensure that included participants were engaged during the task. Acceptable accuracy was defined as a score of greater than 50% correct on the perceptual control task and greater than 40% correct on the “easiest” condition for each of the experimental tasks. The following conditions were considered the easiest: for the grammaticality task the plurality violation condition, for the sound task the rhyming condition and for the meaning task the high association condition. No response bias was indicated by an accuracy difference lower than 40% between the rhyme and unrelated conditions for phonology, the high association and unrelated conditions for semantics, and the plurality violation and the grammatically correct conditions for the grammaticality task.

Acceptable motion was defined as participants having no more than 10% of all volumes or six consecutive volumes being outliers per run. Outlier volumes were volumes exceeding 1.5 mm volume-to-volume head movement in any direction, greater than 5 mm from the mean functional image or the baseline image (first functional image) or deviations of more than 4% from the global mean signal.

#### fMRI data acquisition

Images were acquired using 3T Siemens Skyra MRI scanner with a 64-channel head coil. Functional images were acquired using a susceptibility T2-weighted single-shot EPI method with the following parameters: TR = 1250 ms, TE = 30 ms, FOV = 256 × 256 mm, matrix size = 128 × 128, bandwidth = 1776 Hz/Px, slice thickness = 2 mm without gap, number of slices = 56, voxel size = 2 mm isotropic, ip angle = 80°, multiband acceleration factor = 4. Slices were acquired interleaved from foot-to-head. A high-resolution T1-weighted structural image was also acquired using the following parameters: TR = 1900 ms, TE = 2.43 ms, FOV = 256 × 256 mm, matrix size = 256 × 256, bandwidth = 180 Hz/Px, slice thickness = 1 mm, number of slices = 192, voxel size = 1 mm isotropic, ip angle = 9°.

### 4.4. Data analysis

Preprocessing and analyses were analyzed using Statistical Parametric Mapping, twelfth edition (SPM12) software (https://www.fil.ion.ucl.ac.uk/spm/software/spm12/).

#### fMRI preprocessing and first-level analysis

All functional images were first realigned to their mean functional image across runs. Then, anatomical images were segmented and warped to a pediatric template of children between 7.0–9.0 years old^82^ to obtain the transformation field. This was done by applying an anatomical brain mask created from grey matter, white matter and cerebro-spinal fluid to the original anatomical image to create a skull-stripped image. All functional images and the mean functional image were co-registered to this skull-stripped anatomical image and normalized to the pediatric template using a 6 mm Gaussian kernel. The pediatric tissue probability map template was created using the CerebroMatic toolbox that contains anatomical estimates generated from 1919 participants spanning an age range between 13 months and 75 years. Data were not smoothed for the MVPA analysis.

To reduce motion effects, we used ArtRepair (https://www.nitrc.org/projects/art_repair/) to identify outlier volumes in the functional images ^83^. Outlier volumes were defined as volumes exceeding 1.5 mm volume-to-volume head movement in any direction, head movement greater than 5 mm in any direction from the mean functional image across runs or the baseline image (first functional image), or deviations of more than 4% from the global mean signal. Outlier volumes were replaced with interpolated values from adjacent non-outlier volumes. No more than 10% of volumes in each run and no more than six consecutive volumes for any individual were interpolated in this way as participants with more outlier volumes were excluded from the analyses. These criteria are based on previous publications using the same data^84,85^.

First level statistical analyses were run for the grammaticality (sound task and meaning tasks were only used to create functional localizers) and included ten regressors for each run, one for each of the task conditions and six motion regressors that were estimated in the realignment step. Repaired volumes were de-weighted^83^. All experimental trials (correct and incorrect) were included in the analysis and modeled using a canonical hemodynamic response function. From the first level analyses, the relevant events (finiteness and grammatical condition) were pulled out to use in the MVPA searchlight analyses.

#### Functional regions of interest (ROIs)

The functional regions of interest (ROIs) used for this project here were previously created for the univariate analyses^17^ prior to preregistering the current study design. The inferior frontal gyrus opercularis (IFGop), inferior frontal gyrus triangularis (IFGtri), posterior middle temporal gyrus (pMTG) and posterior superior temporal gyrus (pSTG) were originally chosen based on previous studies showing their relevance for phonologic and semantic processing^86,87^. Within our sample, these regions were identified using the anatomical automatic labeling (AAL) atlas template^88^ using the AFNI – 3dcalc command (https://github.com/afni/afni/blob/master/src/3dcalc.c). The T1 structure of the AAL atlas was warped to pediatric T1 template before selecting the anatomical ROIs. Within the anatomical regions, we created functional localizers for phonology (pSTG and opIFG) and semantics (pMTG and trIFG) based on activation during the sound and meaning task, respectively. For the phonology, the contrast of onset + rhyme > perceptual of the in-scanner sound task was correlated with a behavioral score of phonology (elision of the CTOPP)^89^ and for the meaning task, the contrast of high + low association > perceptual of the in-scanner meaning task was correlated with a behavioral score of semantics (word classes of the CELF-5) ^80^. The Marsbar toolbox^90^ was used to identify the top 500 activated voxels (regardless of significance) from the SPM T-map within the anatomical regions for the contrasts of interest, unless there were voxels with 0 t-value, in which case we selected only the top voxels that could be rank ordered. This resulted in functional ROIs of 500 voxels for the IFGop, pSTG and pMTG and 366 voxels for the IFGtri (given several voxels with t-values of 0). Note that this is a deviation from the preregistration in which we specified to take the top 500 for all ROIs.

#### ROIs – main effect

MVPA searchlight analyses were performed in Matlab using the CoSMoMVPA toolbox (https://www.cosmomvpa.org/index.html)^91^ for each of the functionally localized regions of interest (pSTG, opIFG, pMTG, trIFG). Within the selected top voxels for each of the ROIs, we performed a 25-voxel searchlight analysis using a Support Vector Machine (SVM) classifier to examine spatial patterns of voxels that can reliably distinguish between grammatical sentences and sentences with a finiteness violation. Classification accuracies were obtained by using a leave-one-trial-out cross-validation method. Our data included 20 trials (10 per run), thus the classifier was trained on 19 trials and tested on the remaining one. This procedure was then repeated for the remaining trials until all possible train/test partitions had been exhausted. We obtained the average classification accuracy for the 20 train/test partitions for each of the voxels in the functional ROIs per participant per run. The average classification accuracy matrix obtained for each individual was stacked using the cosmo_stack function^91^ to further perform group-level statistics.

Next, we performed a one-tailed one-sample t-test at the group level across the stacked accuracy matrices to determine the voxels where the classification accuracy was significantly above chance, i.e., above 50%, as our data contained two conditions. The result was corrected for multiple comparisons by submitting the stacked accuracy matrix to a 10,000 iteration Monte Carlo test permutation with Threshold Free Cluster Enhancement (TFCE)^92^ using the cosmo_montecarlo_cluster_stat function in CoSMoMVPA toolbox^91^. TFCE assigned each voxel a “cluster enhanced” value that represents the amount of cluster-like local spatial support based on the spatial extent (mass) and height (peak) of activation while controlling for family-wise error rate^92^. With the cosmo_montecarlo_cluster_stat function, our accuracy matrix was compared against 10,000 matrices with randomized accuracies. Comparing our actual accuracy matrix to these randomized matrices resulted in a new matrix of z-scores for the probability of finding the same or higher classification accuracies as for the randomized data in our actual data. This matrix was then thresholded to a z-score of 1.64, corresponding to a p-value of .05 (one-tailed, as we were only interested in examining accuracies significantly above 50% and not below).

#### ROIs– Correlation with language skill

To examine if language abilities play a role in whether phonological or semantic mechanisms are engaged during morphological processing, we conducted two separate correlation analyses between the accuracy score of the classifier of each voxel and behavioral language abilities as defined by behavioral scores on the Sentence Repetition and the Word Structure tests of the CELF-5. Nonverbal cognitive abilities standard scores were included as a covariate of no interest. Similar to the searchlight analyses, each correlation matrix was corrected for multiple comparisons by submitting the correlation matrix to a 10,000 iterations Monte Carlo test permutation analysis with TFCE^92^ using the cosmo_montecarlo_cluster_stat function^91^. For these iterations, 10,000 “null datasets” with randomized scores were created of the behavioral score (Sentence Repetition or Word Structure), and the covariate of no interest (nonverbal cognitive abilities standard scores). Thereafter, 10,000 null correlation matrices were computed between classification accuracies and null datasets of language abilities (Sentence Repetition or Word Structure) while controlling for nonverbal cognitive abilities. Voxel-wise correlation values were compared to null datasets spread around a baseline of 0 using the cosmo_montecarlo_cluster_stat. This differs from the searchlight approach as correlation values rather than z-values were submitted to cosmo_montecarlo_cluster_stat. This procedure gives values between 0 to 1 for each voxel, with a value between 0 and 0.5 indicating that the TFCE value was negative and a value between 0.5 and 1 that the TFCE value was positive. These values were computed into a z-score using the inverse normal cumulative distribution function (cosmo_norminv). The z-scores for each voxel indicated the probability to find the same, or higher, TFCE value under the null hypothesis of one-sample t-test against 0.05. TFCE-corrected maps were thresholded voxel-wise at z ≤ −1.64 and z ≥ 1.64, corresponding to p < .05 after correction for multiple comparisons.

#### Whole brain – Correlation with language skill

To explore whole brain correlations of the classification accuracy scores for distinguishing between grammatical sentences and sentences with a morphological finiteness violation with language skill, correlation analyses were performed using the same approach as in the ROIs but using a 100-voxel searchlight rather than a 25 voxel one. The TFCE correction for multiple comparisons approach was the same as for the ROI correlation.

## Supplementary Material

Supplementary Files

This is a list of supplementary files associated with this preprint. Click to download.

• ScientificReportsMVPAMuesSupplementarymaterials.docx

## Figures and Tables

**Table 1:** Behavioral performance on language tasks

	M	SD
**Standardized Language Measures**		
Elision test^1^	11.4	2.8
Word Classes test^1^	12.5	3.2
Sentence Repetition test^1^	11.6	3.0
Word Structure test^1^	10.3	2.5
**In-scanner Sentence Processing task**		
Grammatical sentences^2^	80.9	14.9
Finiteness violation condition^2^	70.9	21.7
Plurality violation condition^2^	87.4	12.8
Perceptual control condition^2^	95.7	8.0

1 Scaled scores (population mean of 10, SD of ± 3)

2 Percentages

**Table 2.** Summary of negative correlations with Sentence Repetition after TFCE corrections

*Location*	*Number of voxels (k) combined*	*Average z- value*	*MNI coordinates of largest cluster*		
			x	y	z
**LEFT HEMISPHERE**									
*Frontal lobe*									
L. IFG Triangularis	120	−2.04	−36	38	20
L. Middle Frontal Gyrus	77	−2.05	−30	46	34
L. Precentral Gyrus	10	−2.06	−48	4	42
L. Superior Medial Gyrus	14	−2.06	−14	32	34
*Parietal lobe*					
L. Angular Gyrus	14	−2.61	−42	−60	46
L. Inferior Parietal Lobe	12	−2.08	−34	−54	48
L. Postcentral Gyrus	16	−2.25	−46	−26	54
*Temporal lobe*					
L. Middle Temporal Gyrus	11	−2.09	−52	−54	8
*Occipital lobe*					
L. Calcarine Gyrus	10	−2.34	2	−70	24
**RIGHT HEMISPERE**					
*Frontal lobe*					
No ^3^ *k* = 10					
*Parietal lobe*					
R. Rolandic Operculum	10	−1.96	52	−10	14
*Temporal lobe*					
No ^3^ *k* = 10					
*Occipital lobe*					
R. Calcarine Gyrus	33	−2.00	12	−74	14
R. Lingual Gyrus	19	−2.00	12	−72	0

**Note.** Table shows all maxima separated by more than 20 millimeters. Regions were automatically labeled using the SPM Anatomy Toolbox via bspmview.

**Table 3:** Summary of negative correlations with Word Structure after TFCE corrections

*Location*	*Number of voxels (k) combined*	*Average z- value*	*MNI coordinates of largest cluster*		
			x	y	z
**LEFT HEMISPHERE**					
*Frontal lobe*					
L. Middle Frontal Gyrus	17	−2.42	−42	14	50
*Parietal lobe*					
No ≥ *k* = 10					
*Temporal lobe*					
L. Middle Temporal Gyrus	35	−2.32	−52	−46	10
L. Superior Temporal Gyrus	16	−2.07	−62	−52	22
*Occipital lobe*					
L. Calcarine Gyrus	12	−2.48	−22	−62	12
L. Lingual Gyrus	12	−2.79	0	−76	6
**RIGHT HEMISPHERE**					
*Frontal lobe*					
R. Anterior Cingulate	24	−1.80	10	44	20
R. IFG Opercularis	28	−1.91	42	10	18
R. IFG Triangularis	93	−1.93	46	22	10
R. Middle Frontal Gyrus	79	−1.91	32	16	48
R. Precentral Gyrus	65	−2.08	40	−6	46
R. Superior Frontal Gyrus	16	−1.80	20	4	56
R. Superior Medial Gyrus	21	−1.77	12	24	46
*Parietal lobe*					
R. Angular Gyrus	45	−2.02	42	−66	42
R. Inferior Parietal Lobule	26	−2.02	48	−48	52
R. Rolandic Operculum	37	−1.86	52	2	22
*Temporal lobe*											
R. Middle Temporal Gyrus	22	−2.07	46	−70	26
R. Postcentral Gyrus	58	−1.98	52	−16	36
R. Superior Temporal Gyrus	10	−2.07	46	−28	8
R. Supramarginal Gyrus	20	−2.04	48	−38	36
*Occipital lobe*											
R. Calcarine Gyrus	61	−2.10	22	−72	10
R. Lingual Gyrus	34	−2.09	8	−80	2
R. Middle Occipital Gyrus	28	−2.05	32	−70	44
*Other*											
R. Insula	36	−1.99	38	−14	4
R. Midcingulate cortex	59	−2.02	10	28	36
R. Palladium	18	−2.36	28	−10	4
R. Putamen	16	−2.32	30	0	16

**Note.** Table shows all maxima separated by more than 20 millimeters. Regions were automatically labeled using the SPM Anatomy Toolbox via bspmview.

**Table 4:** Examples of grammatically task.

Condition	Example	Description
Grammatically correct	She is moving the box	No grammatical violation
Finiteness violation	Every day, she **press** one button*	A morphological violation on the verb form.
Plurality violation	Every day, they stir three pot*	A violation in number and object agreement
Perceptual control	Shh-shh	Frequency modulated noise

**Table 5:** Examples of phonology and semantic task.

Condition	Example	Description
**Sound task**		
Rhyme condition	Wide-Ride	Two words that share the coda
Onset condition	Coat-Cup	Two words that share the first sound
Unrelated condition	Zip-Cone	Two words that do not share sounds
Perceptual control	Shh-shh	Frequency modulated noise
**Meaning task**		
High association	Water-Drink	Two words with a strong semantic association
Low association	Syrup-Pancake	Two words with a weak semantic association
Unrelated condition	Flush-Cliff	Two words with no semantic association
Perceptual control	Shh-shh	Frequency modulated noise

## Data Availability

The data necessary to reproduce the analyses presented here are publicly accessible on OpenNeuro via https:/openneuro.org/datasets/ds003604/versions/1.0.7 . The CoSMoMVPA scripts to reproduce the searchlight analyses can be found here: https:/github.com/avantika9/ELP_MVPA . The analyses presented here were preregistered on Open Science Framework: https://osf.io/6vdaw.
